# Role of imaging in the management of neuro-ophthalmic disorders

**DOI:** 10.4103/0301-4738.77015

**Published:** 2011

**Authors:** Rashmin Anilkumar Gandhi, Akshay Gopinathan Nair

**Affiliations:** Sankara Nethralaya, A Unit of Medical Research Foundation, Chennai - 600 006, Tamil Nadu, India

**Keywords:** Computed tomography, CT angiography, magnetic resonance imaging, MR angiography

## Abstract

Advancements in physics, computers, and imaging science in the last century have seen neuro-imaging evolving from a plain X-ray to computed tomography, magnetic resonance imaging scans, noninvasive angiography, and special sequences such as fat suppression, fluid attenuation recovery and diffusion-weighted imaging. A prompt prescription of an appropriate imaging modality and the most suitable sequence can increase the diagnostic yield, and in many instances, it can be a sight-saving and even a life-saving decision. This article discusses basic principles of neuro-imaging, its common indications, and the appropriate application in an ophthalmology practice.

Radio-imaging has come a long way from the discovery of X-rays in 1895 to the development of sophisticated imaging techniques such as computed tomography (CT) and magnetic resonance imaging (MRI). Being able to image the internal structures of the body with precision has changed the diagnostic and therapeutic protocols of diseases in every specialty of medicine, and ophthalmology is no exception. Very often, the ophthalmologist may be the first clinician to come in contact with a patient with symptoms suggestive of an intracranial tumour or an infarct and the situation would warrant appropriate and in some instances urgent neuro-imaging. Hence, it is essential for an ophthalmologist to know exactly which mode of imaging should be asked for, the additional benefits of contrast agents, and more specifically, the interpretation of the myriad images generated by the imaging studies. In this article, we have summarized the role of neuro-imaging according to neuro-ophthalmic symptoms. We wish to emphasize that although neuro-imaging may help in localizing a lesion, radiological investigations should be ordered judiciously and can never replace a thorough clinical examination.

Common indications of neuro-imaging covered here are visual impairment, fundus abnormalities, hemifacial spasm, and ocular motility disorders.

## Visual Impairment

### Acute loss of vision: Unilateral

In patients with acute unilateral loss of vision due to the visual pathway disorder, the areas of interest for the ophthalmologists are the optic nerve and the optic chiasm. Magnetic resonance imaging (MRI) is the examination of choice here because it provides superior soft-tissue discrimination of intracranial anatomy.[1]

A brief explanation of physics will help us understand the superiority of MRI in acquiring soft tissue details. The MR signal is generated by the differences in relaxation times of protons in hydrogen which are present in water molecules, after being subjected to a magnetic field and a specific radiofrequency (RF) pulse. The MR scanner induces and detects the resonance of small magnetic fields generated by atomic nuclei. Normally (in the absence of the magnetic field), the axes of these small magnetic fields are randomly arranged. When placed in the external magnetic field of the MR scan, these small magnets align themselves with the axis of the applied field. After the RF pulse is applied, some of this energy is absorbed by the nuclei, and they orient against the magnetic field. On turning the RF pulse off, the nuclei which are now at a higher energy level release the absorbed energy and return to their original states and alignments. This release of energy can be picked up by the scanner as a detectable signal. This process is called “relaxation”. The rate of change and the intensity of the signal depend upon the number of nuclei involved and the inherent relaxation characteristics of the surrounding chemical environment. These characteristic relaxation times are the parameters of MR.[2] T1 (spin-lattice relaxation time) is the longitudinal relaxation time. It indicates the time required for a substance to become magnetized after first being placed in a magnetic field or, alternatively, the time required to regain longitudinal magnetization following an RF pulse. Adjacent spinning protons may have different spinning velocities, and the resultant interaction can produce changes in the magnetic moments of these adjacent spinning protons. These protons exchange spin with their neighboring protons.

**Figure 1:**
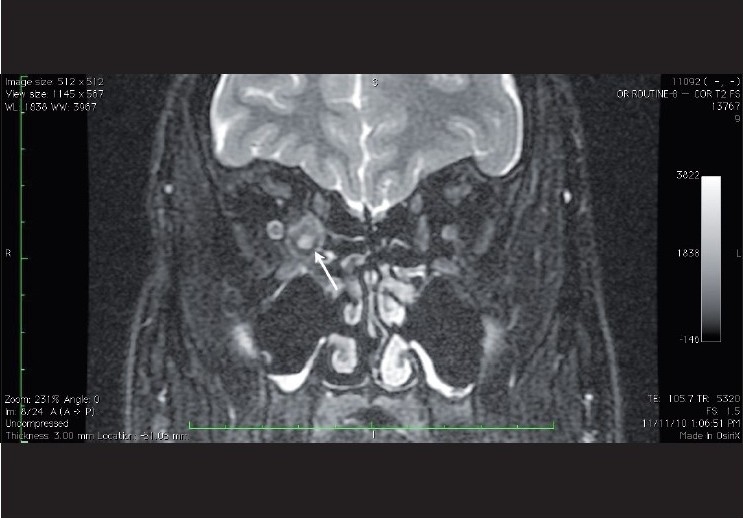
Coronal Fat suppressed T2 weighted image showing the right medial rectus muscle with a well defined rounded cystic lesion with an eccentric scolex within (white arrow), suggestive of cysticercosis.

**Figure 2:**
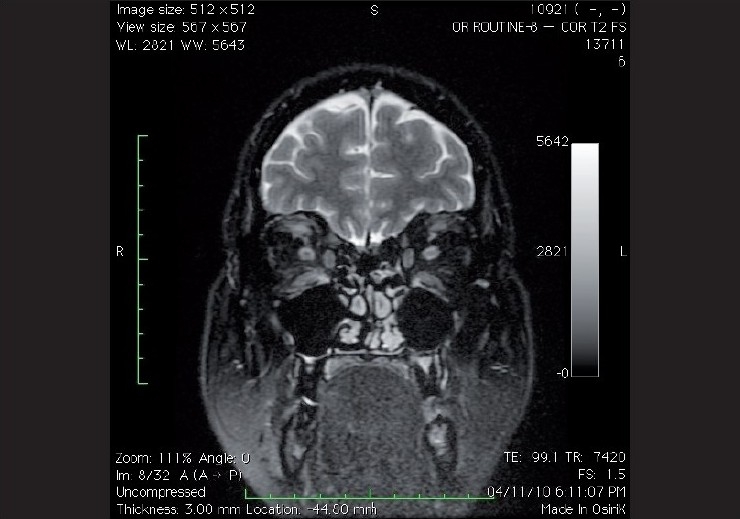
Coronal fat supressed T2 weighted image showing thickened extra ocular muscles bilaterally. Note the hyperintense signals in the bilateral inferior recti muscles and the left superior rectus suggestive of active thyroid eye disease

Spin-spin relaxation time (T2) refers to the time that is taken for a loss of signal because of the aforementioned spin interactions of adjacent nuclei.[2] Unlike T1 interactions, T2 interactions do not involve any transfer of energy but only a change in the phase, which leads to a loss of coherence. Thus, the relaxation times can be varied thereby resulting in changes in the contrast of images; and the study can be “weighted” toward a T1 or T2 study. *Normal anatomy is best demonstrated on T1-weighted images, where as T2-weighted images are typically better for demonstrating intracranial or other pathology* [Figs. 1 and 2].[1,2]

**Figure 3:**
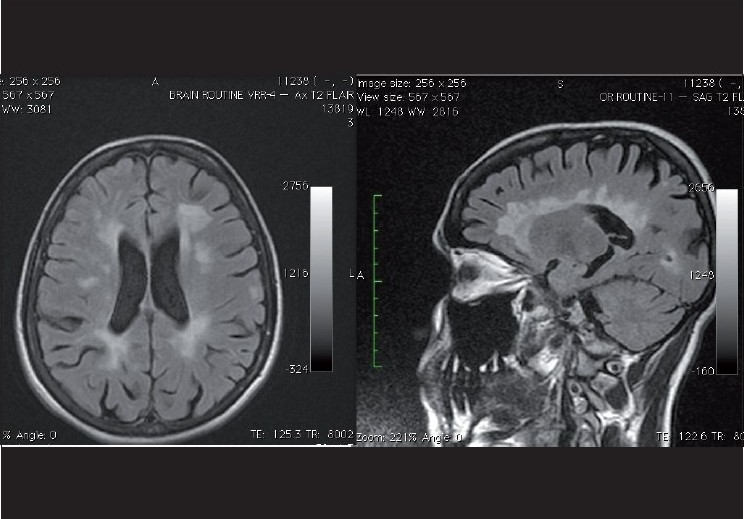
Left: Axial T2 FLAIR image at the level of the lateral venticles showing multiple hyperintense periventricular white lesions, suggestive of Multiple Sclerosis. Right: Para sagittal T2 FLAIR image showing multiple hyperintense periventricular white lesions perpendicular to the lateral ventricles, also known as Dawsons fingers

The role of MRI is vital in cases of demyelinating optic neuritis. It is equally important in evaluating the potential risk of future demyelinating events and multiple sclerosis (MS). The optic neuritis study group, after a 15-year follow-up, has shown that patients with optic neuritis and no white matter lesions on initial MRI had a 25% risk of developing MS, while those with one or more lesions had a 72% risk [Fig. 3].[3,4]

On imaging, in the acute phase, fat saturated T2-weighted and short TI inversion recovery (STIR) images typically show swelling and increased signal intensity of the optic nerve and contrast-enhanced fat saturated T1-weighted images show enhancement of the nerve itself.

**Figure 4:**
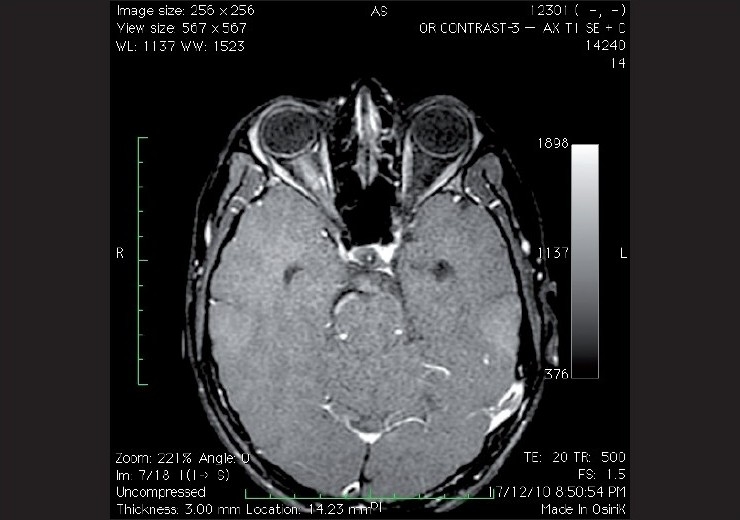
Contrast-enhanced axial T1 weighted image with fat suppression shows enhancement and thickening of perineural structures of right optic nerve, suggestive of optic neuritis

FLAIR (fluid-attenuation inversion recovery) sequences help reveal demyelination or MS plaques in the central nervous system, tumours, and ischemic lesions that often are not visible on routine MRI imaging. Here, as the name suggests, the cerebrospinal fluid (CSF) signal is strongly attenuated, accentuating periventricular and extra-axial disease near the brain surface,[5] thereby allowing visualization of the underlying pathology (e.g., white matter demyelination, posterior reversible encephalopathy) that might otherwise be obscured by the bright signal of normal CSF [Fig. 4].[6]

To summarize, in patients with acute loss of vision, MRI scan of the brain and orbit is the imaging modality of choice. Orbital fat suppression provides better details of optic nerve enhancement.

**Figure 5:**
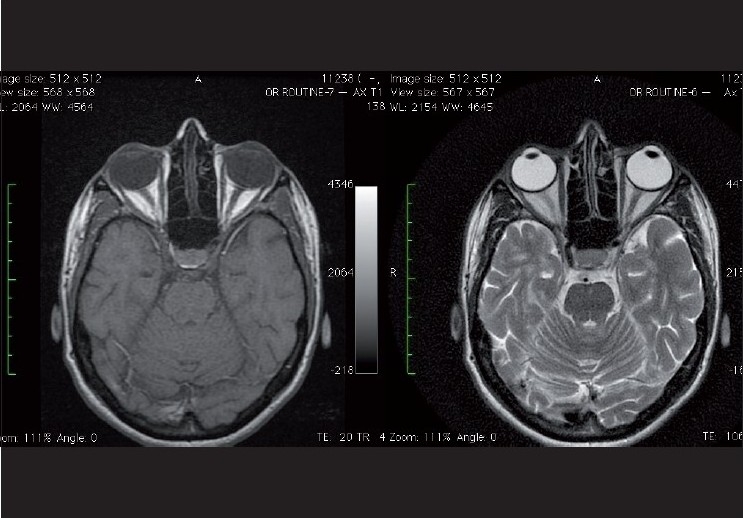
Left: Axial T1 weighted image showing the normal optic nerve and the characteristic hyperintense appearing orbital fat surrounding it. Right: Axial T2 FSE (Fast Spin Echo) image depicting the normal optic nerve. Note the bright hyperintense cerebrospinal fluid

It may help the uninitiated ophthalmologist to remember that fat appears bright in T1 images (hyperintense) and CSF appears hyperintense in T2 images [Fig. 5].

**Figure 6:**
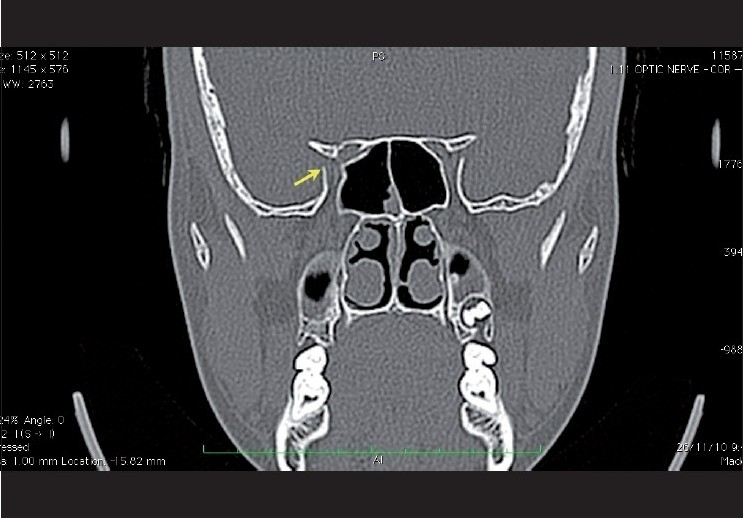
Coronal CT scan of the orbit at 1mm interval for the optic canals in the bone window setting showing a fracture of the medial wall and the roof of the right optic canal (Yellow arrow)

In patients with suspected traumatic optic neuropathy, a CT scan of the brain with fine cuts (axial sections of 1-1.5 mm) through the orbits should be sought. Coronal images are necessary to evaluate the optic canal properly and to rule out a fracture. In patients with traumatic optic neuropathy, orbital fractures especially canal fractures have been associated with poorer visual acuity and a poor prognosis [Fig. 6]. MRI of the orbit may reveal focal edema of the optic nerve or optic nerve sheath enhancement with gadolinium.

Intravenous contrast material improves detection of pathology by demonstrating areas of blood-brain barrier breakdown. Iodinated contrast agents are used in imaging techniques based on X-rays, as these agents are radio-opaque which means they directly absorb or deflect photons from the X-ray beam.[2] The contrast material commonly used in MRI is gadolinium, a paramagnetic substance, that remains extracellular and is excreted renally. Gadolinium is a non-radio-opaque material and hence is not of use in X-ray-based techniques. The gadolinium paramagnetic metal ion enhances the local magnetic field and increases signal intensity and is generally used for T1-weighted imaging [Fig. 4].[1,7]

### Acute loss or disturbance of vision: Bilateral

In such cases the areas of interest are the chiasmal and post-chiasmal visual pathway. Common cause for an acute bilateral diminution of vision is ischemia of the post-chiasmal visual pathway. Here, the imaging mode of choice is an MRI. A special sequence to look for would be diffusion-weighted imaging (DWI).

Water molecules tend to be in a constant state of random motion called Brownian movement. The rate of movement of the water molecules or diffusion depends on the kinetic energy of the molecules and also the temperature. In biological tissues, diffusion is not truly random because tissues for example, cell membranes have a definite structure which may restrict the amount of diffusion. Also, chemical interactions of water and macromolecules affect diffusion properties. This water mobility, in the brain, is referred to as “apparent diffusion co-efficient” (ADC). The combination of DWI/ADC can help define specific pathology of water diffusion. In pathological conditions, this diffusion becomes restricted and DWI can help to differentiate between the various phases of cerebral infarction.[1] Only the acute infarcts appear hyperintense on the diffusion images. DWI can detect hyperacute ischemic stroke even before abnormalities are detected on conventional T1-weighted images, T2-weighted images, and FLAIR sequences. While suspecting an acute infarct, the need for DWI may have to be communicated to the radiologist as it may not be routinely done during MRI of the head at all institutes.

### Gradually progressive loss of vision: Unilateral

The role of neuro-imaging in a patient with gradual progressive loss of vision is to diagnose or rule out a compressive lesion in the orbit. Causes of compressive optic neuropathy include thyroid ophthalmopathy, optic nerve and orbital tumours, inflammatory infiltrative processes, mucoceles, pituitary macroadenomas, meningiomas arising from the diaphragma sellae and craniopharyngiomas.[8] Common tumours include optic nerve glioma, the commonest primary tumour of the optic nerve or an optic nerve meningioma, which is the most common primary tumour arising from the optic nerve sheath.

In cases where an orbital tumour is suspected, a CT scan can show the tumour mass clearly, especially if better delineation of bony anatomy is required. Although in some cases where the tumour may extend intracranially, an MRI may be needed to map out the entire extent of the tumour. Specific tumour features such as calcification or hyperosteosis (seen commonly in meningiomas) are also better picked up on a CT scan.

CT is based on the principle of X-ray attenuation by different tissues possessing different densities. It involves the rapid rotation of a large X-ray tube around the patient. The presence of X-rays, obviously mean radiation exposure to the patient in a CT scan as opposed to an MRI scan which involves no radiation exposure.

The resultant transmitted radiation is measured by a ring of detectors, and the data collected by the detectors are analysed by a computer. The density, or attenuation value, of the tissue at each point is calculated and an image is reconstructed as a corresponding matrix of picture elements which are called pixels, with each pixel being assigned a numerical value.[1] These values are then compared with the attenuation value of water and displayed on a scale of arbitrary units called Hounsfield units; named after Sir Gordfrey Hounsfield, the pioneer of CT. The Hounsfield scale is a quantitative scale for describing radiodensity. This scale assigns water as an attenuation value of zero. Denser material (e.g., lead) blocks or attenuates the X-ray beam, and less dense material allows the beam to pass through to the detectors. Thus, dense materials such as bone appear brighter (positive Hounsfield units) on CT and less dense material like air appear darker (negative Hounsfield units).[1]

In thyroid eye disease, MRI can distinguish acute inflammatory active disease from fibrotic, inactive end-stage disease in demonstrating interstitial edema within the extraocular muscles [Fig. 2]. MRI is also the modality of choice to identify active inflammatory changes and to assess any immunomodulatory treatment response.[9] Although, if a bony compressive pathology is being suspected, a CT scan may show bony anatomy better.

In a carotid-cavernous fistula, CT or MR angiography may be used, though catheter angiography offers the option of endovascular treatment. The classical feature to look out for is the enlargement of the ipsilateral cavernous sinus and a distended ipsilateral superior ophthalmic vein.[8]

**Figure 7:**
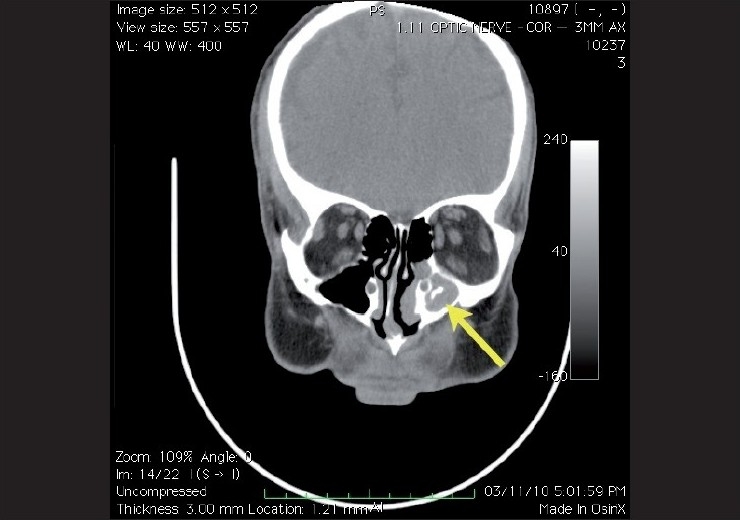
Coronal CT scan of the orbit at the mid orbit level showing normal optic nerves and extra ocular muscles. Note the left maxillary sinusitis (yellow arrow)

When ordering a CT scan, it is essential that the ophthalmologist specify whether the investigation includes the head or the orbit or both. An orbital CT scan differs from a head CT in that the images are typically obtained at a different angle using thinner slices (e.g., as thin as 0.7 mm *vs*. 3–4 mm) and can be reconstructed to any slice thickness [Fig. 7].[1]

### Gradually progressive loss of vision: Bilateral

Here, the areas of interest will be the optic chiasm and the post-chiasmal pathways, and the pathology to look for is compressive lesions arising from pituitary gland. Pituitary tumours are classified as microadenomas (size < 10 mm) or macroadenomas (size > 10 mm) depending on their size. MR imaging of the position of the intracerebral optic nerves, optic chiasm, and optic tracts is of utmost importance for surgical planning and is best assessed with high-resolution T2-weighted and contrast-enhanced T1-weighted images.[8]

## Fundus Findings

Sometimes, fundus findings may necessitate the need for imaging. An elevated disc may be seen unilaterally in an optic nerve head drusen, nonarteritic ischemic optic neuropathy, and rarely in infiltrative optic neuropathy.

Optic nerve head drusen are white calcareous deposits, seen either superficially on the optic nerve head or buried within it.[10] They occur possibly due to axonal degeneration of the optic nerve. They may mimic the appearance of papilledema. However, unless they are large enough to compress nerve fibers and subsequently cause vascular occlusions, they usually need only regular monitoring. Although a B-scan ultrasonography (USG) can effectively document the lesion; if need be, a CT scan with thin cuts of 1 mm each may be ordered in order to demonstrate the lesion and the calcification within.[8]

When encountered with nonarteritic ischemic optic neuropathy (NAION), MRI with contrast enhancement may help differentiate NAION, from optic neuritis on the basis of their different contrast enhancement patterns. Infiltrative optic neuropathies, apart from MRI to clearly demonstrate the neural infiltration, may also need additional invasive diagnostic procedures such as a CSF examination to know the exact cause of the disease.[11]

**Figure 8:**
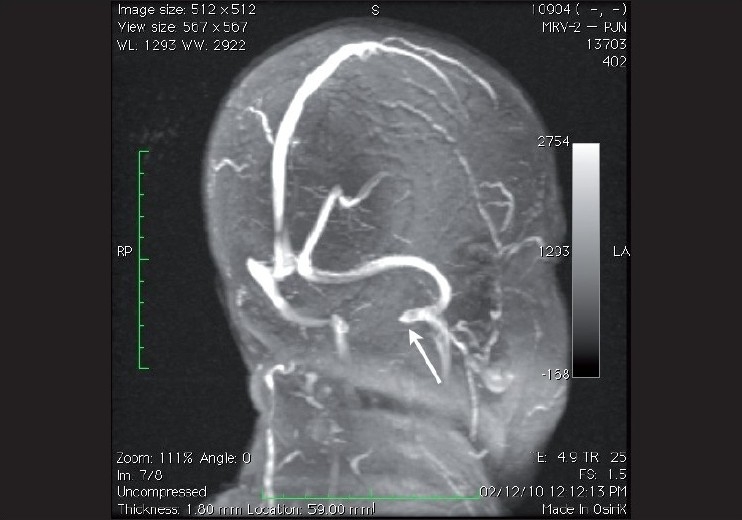
Oblique MIP (Maximum Intensity projection) of 2D time of flight venogram showing normal superior sagittal sinus, transverse sinus, sigmoid sinus and the jugular bulb (white arrow)

Bilaterally elevated discs may result from a raised intracranial pressure. It is essential to rule out the presence of an intracranial tumour or a dural sinus thrombosis prior to diagnosing the condition as idiopathic intracranial hypertension (IIH). Benign IIH is a common cause of papilledema in young, overweight females. Early onset of medical treatment has prevented them from ending up with irreversible vision loss and optic atrophy. The investigative modality of choice in IIH is MRI + MR venogram [Fig. 8].[12]

## Hemifacial Spasm

Hemifacial spasm is caused by pulsatile vascular compression upon the facial nerve root exit zone. This 2–3 mm area is a transition zone between central and peripheral axonal myelination that is situated at the nerve’s detachment from the pons.[13] MR imaging, with emphasis on the posterior cranial fossa, can clearly chart the course of the seventh nerve from the root exit zone of the brainstem to the internal auditory canal and its relationship to the surrounding vertebrobasilar system and demonstrate any vascular occlusion, if present.[14]

## Ocular Motility Disorders

Cranial nerve palsies form yet another group of disorders which may require neuro-imaging to identify the etiology. III nerve palsies present the neuro ophthalmologist yet another dilemma when it comes to ordering neuro-imaging. While a large proportion of cases of III nerve palsies are ischemic (i.e., diabetes and hypertension), a significant minority of cases are secondary to aneurysm, the most feared cause of III nerve palsies.[15] Combination of urgent MRI/MR-angiography or CT/CT-angiography is advised when assessing a patient with a pupil involving third nerve palsy.[1]

**Figure 9:**
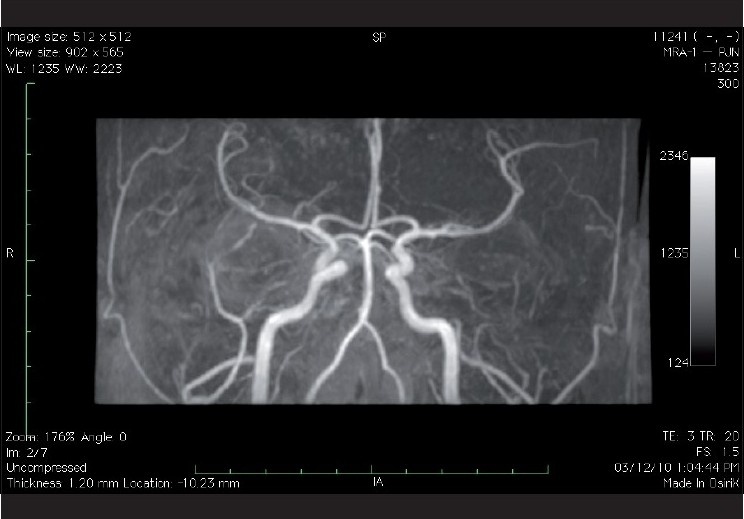
Coronal MIP (Maximum Intensity Projection) of 3D time of flight angiogram showing normal carotid and vertebral angiogram

CT-angiography involves the assessment of the arteries after an injection of an iodinated contrast agent whereas an MR angiography can be performed without any contrast agent [Fig. 9]. As is the case for most ocular motor nerve palsies, patients who have a positive history of vasculopathic risk factors, usually show recovery in the period following the initial attack. If there in a lack of improvement or recovery of the condition, neuro-imaging is advised. However, as the “gold standard” for detecting cerebral aneurysms remains catheter angiography, we recommend its consideration for highly suspicious cases, even in the presence of negative MR angiography or CT angiography.[1]

**Figure 10:**
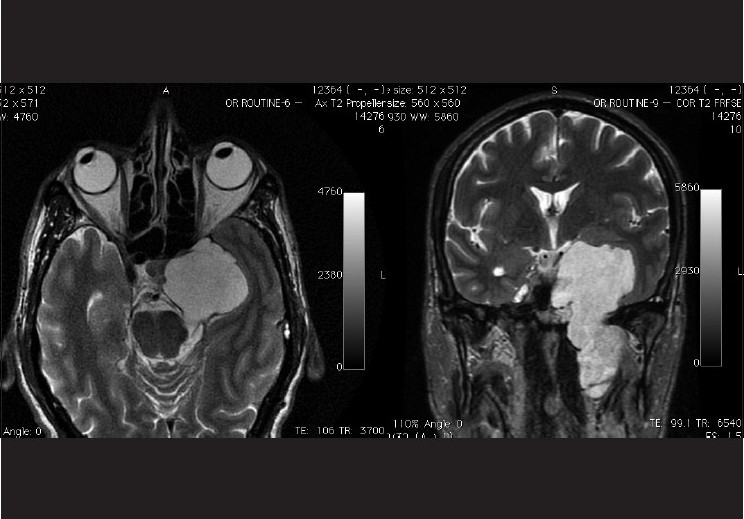
A 54 year old non diabetic, non hypertensive male; who presented with a 6 month old history of left sided VI nerve palsy. Axial T2 weighted image (left) and coronal T2 weighted FRFSE (fast relaxation fast spin echo) image (right) showing a large fairly well circumscribed lobulated heterogenous mass in the left cavernous sinus, displaying a heterogenous hyperintense signal. Note that the cavernous internal carotid artery is not seen separately on the left side

VI nerve palsy is far more common than III N palsy. It may not be feasible to order neuro-imaging for every patient of VI nerve palsy. Current recommendation is to observe cases of isolated VI nerve palsy with vasculopathic risk factors, and to obtain neuro-imaging upon follow-up only if the ophthalmoplegia does not improve, progresses, or becomes nonisolated [Fig. 10].[16] However, VI nerve palsy in younger patients and those without a history of diabetes, hypertension, or trauma must be imaged.

## Conclusion

While there exists a vast variety of neuro-imaging modalities, it is essential that the ophthalmologist orders the right investigation for the condition. MRI has a distinct advantage over other imaging techniques owing to its greater soft tissue differentiating capabilities; however, CT scans are very useful in detecting subtle bony erosions, fractures, and deformities. The key to the correct diagnosis is in tailoring the appropriate investigation for the patient’s condition and communication with the radiologist.
